# Optimization of Anti-SARS-CoV-2 Neutralizing Antibody Therapies: Roadmap to Improve Clinical Effectiveness and Implementation

**DOI:** 10.3389/fmedt.2022.867982

**Published:** 2022-03-28

**Authors:** Karlijn van der Straten, Marit J. van Gils, Steven W. de Taeye, Godelieve J. de Bree

**Affiliations:** ^1^Department of Medical Microbiology and Infection Prevention, Amsterdam Institute for Infection and Immunity, Amsterdam UMC, University of Amsterdam, Amsterdam, Netherlands; ^2^Department of Internal Medicine, Amsterdam Institute for Infection and Immunity, Amsterdam UMC, University of Amsterdam, Amsterdam, Netherlands

**Keywords:** SARS-CoV-2, monoclonal antibodies, variants of concern (VOCs), neutralization, bi-specific antibodies, Fc domain modifications, long-acting therapy, nanobodies (VHH)

## Abstract

One of the major breakthroughs to combat the current Coronavirus Disease 2019 (COVID-19) pandemic has been the development of highly effective vaccines against the Severe Acute Respiratory Syndrome Coronavirus 2 (SARS-CoV-2). Still, alternatives are needed for individuals who are at high risk of developing severe COVID-19 and are not protected by vaccination. Monoclonal antibodies against the spike protein of SARS-CoV-2 have been shown to be effective as prophylaxis and treatment against COVID-19. However, the emergence of variants of concern (VOCs) challenges the efficacy of antibody therapies. This review describes the neutralization resistance of the clinically-approved monoclonal antibody therapies against the Alpha (B.1.1.7), Beta (B.1.351), Gamma (P1), Delta (B.1.617.2), and the Omicron (B.1.1.529) variants. To guide the development of monoclonal antibody therapies and to anticipate on the continuous evolution of SARS-CoV-2, we highlight different strategies to broaden the antibody activity by targeting more conserved epitopes and/or simultaneously targeting multiple sites of vulnerability of the virus. This review further describes the contribution of antibody Fc effector functions to optimize the antibody efficacy. In addition, the main route of SARS-CoV-2 antibody administration is currently intravenously and dictates a monthly injection when used as prophylactic. Therefore, we discusses the concept of long-acting antibodies (LAABs) and non-intravenously routes of antibody administration in order to broaden the clinical applicability of antibody therapies.

## Introduction

Since the beginning of the Coronavirus Disease 2019 (COVID-19) pandemic, over 437 million infections and 5.9 million COVID-19 related deaths are registered worldwide (1). Although the majority of COVID-19 patients develop mild respiratory symptoms or remain asymptomatic, there is a substantial group at risk for severe COVID-19 disease (2). These risk groups include elderly (>65 years of age) and individuals with comorbidities such as obesity (BMI > 30), cardiovascular disease, diabetes, chronic pulmonary disease, and immune-compromised individuals (3). Vaccinations against the causative Severe Acute Respiratory Syndrome Coronavirus 2 (SARS-CoV-2) substantially reduces the risk of severe COVID-19 (4). Therefore, the COVID-19 vaccination campaigns considerably diminished the enormous burden on health care systems worldwide. However, there are still many people not protected against this high risk by vaccination. For those patients with mild to moderate COVID-19 but with a high risk to develop severe COVID-19, five monoclonal antibody therapies received Emergency Use Authorization (EUA) by the FDA and/or EMA to prevent worsening of disease and/or as post-exposure prophylaxis (5–9). In this review, we focus on these anti-SARS-CoV-2 monoclonal antibody therapies and discuss the challenges that hinder their widespread utilization.

Since the beginning of the pandemic, thousands of monoclonal antibodies derived from B cells of convalescent COVID-19 patients have been screened for their binding properties to the spike (S) glycoprotein on the viral membrane and their neutralization capacity to prevent infection *in vitro*. The S protein is the most studied structural protein of SARS-CoV-2 due to its pivotal role in viral attachment to the human cell (10, 11). The S protein is a homotrimeric glycoprotein, with each monomer containing two subunits. The proximal S2-subunit is embedded in the viral membrane and includes the fusion peptide. The other subunit is the distal S1-subunit that contains an N-terminal domain (NTD) and a receptor-binding domain (RBD). The RBDs can adopt either a closed or an open confirmation. Only the open conformation enables interaction between the receptor-binding motif (RBM) within the RBD and the human Angiotensin-Converting Enzyme-2 (ACE-2) receptor (12). Blocking this essential interaction between the ACE-2 receptor and the RBM is the main mechanism of action of SARS-CoV-2 neutralizing antibodies. Accordingly, the most potent neutralizing antibodies isolated target epitopes within the RBD (13, 14). SARS-CoV-2 challenge studies in animals revealed that the neutralization potency of antibodies correlates with protection against COVID-19 (15, 16). Predictive models build upon human clinical data (17) and COVID-19 vaccination studies (18, 19) further confirmed this association. As a result, the neutralizing potency of monoclonal antibodies *in vitro* is often used to predict efficacy *in vivo*.

**Figure 1:**
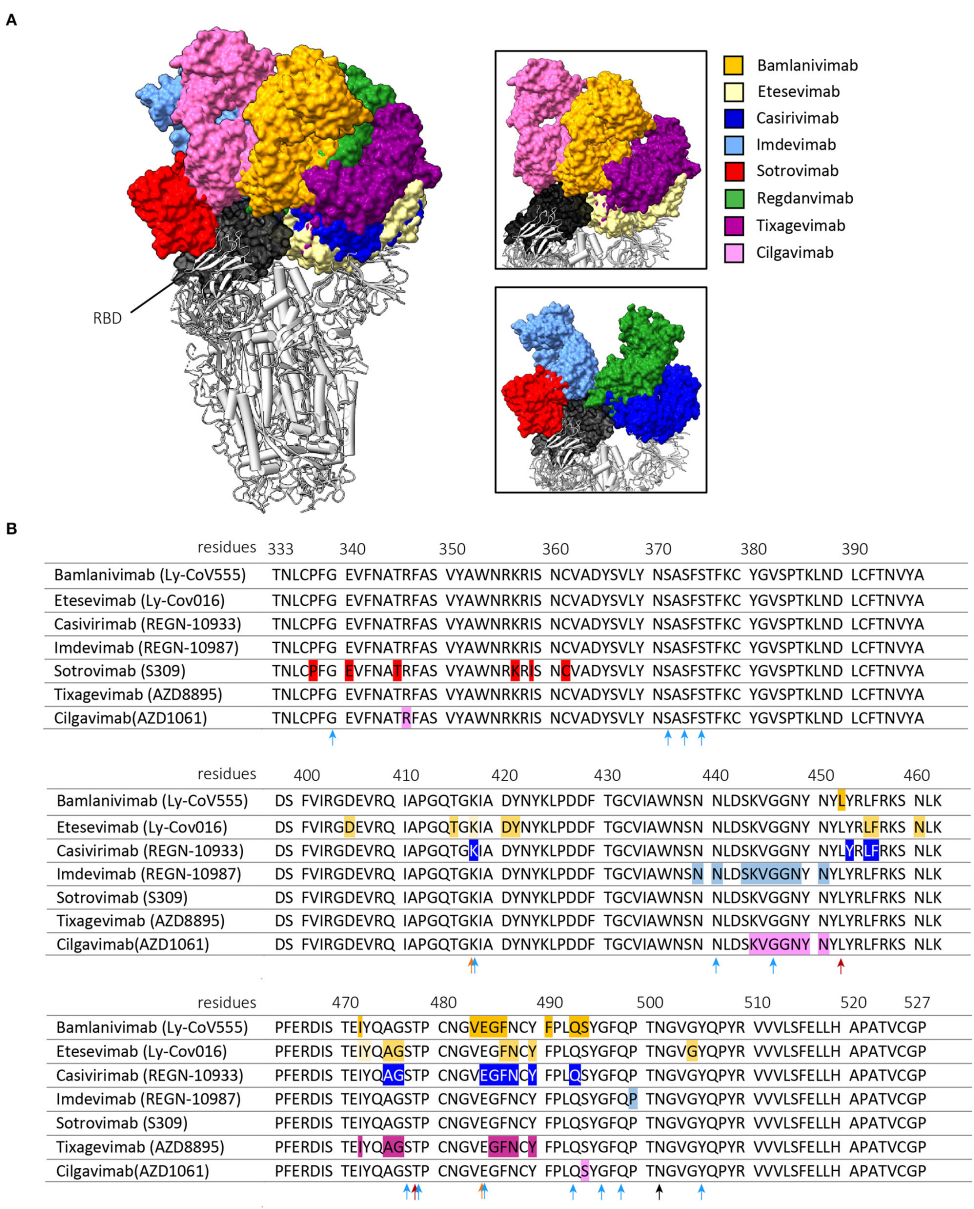
Interaction of the clinically approved anti-SARS-CoV-2 monoclonal antibodies with the RBD of the SARS-CoV-2 S protein. **(A)** shows the binding of the Fragment antigen-binding regions (Fabs regions) of bamlanivimab (PDB: 7KMG), etesevimab (PDB: 7C01) casirivimab (PDB: 6XDG), imdevimab (PDB: 6XDG), sotrovimab (PDB: 6WPT), tixagevimab (PDB: 7L7E), cilgavimab (PDB: 7L7E) and regdanvimab (PDB: 7CM4). The right panels are zoomed in on the interaction between the Fabs and the RBD of the S protein. These figures were made with ChimeraX, version 1.3. **(B)** shows amino acid mutations in the Wuhan-Hu-1 reference sequence (GeneID: 43740578) that result in antigenic escape of the listed monoclonal antibodies. The arrows indicate RBD mutations harbored by the Beta and Gamma variants (orange), the Delta variant (red) or the Omicron variant (light blue). The N501Y mutation is shared by all variants and is indicated by a black arrow. Antigenic escape data is obtained from Bloom et al. (43).

**Figure 2:**
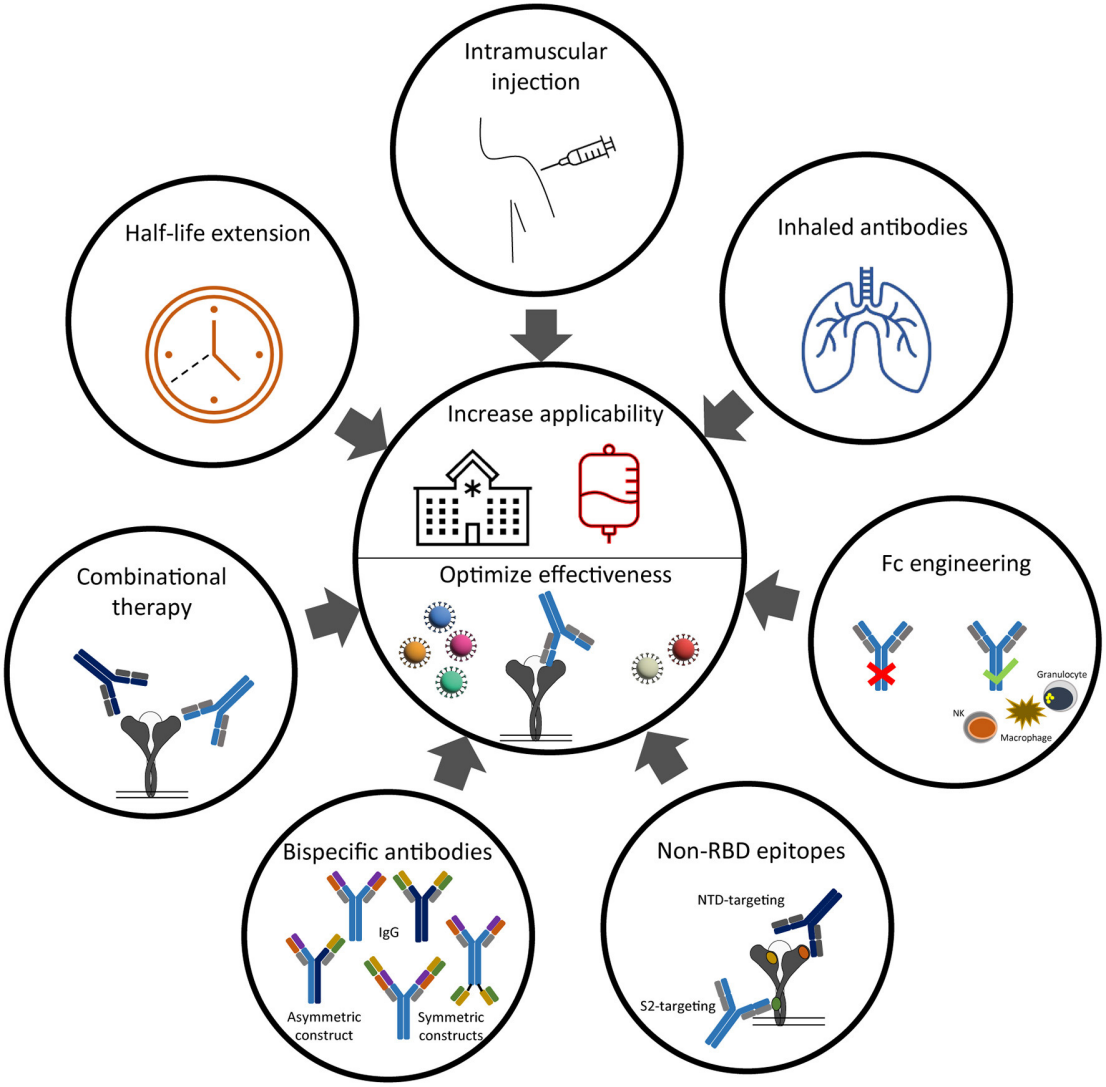
Strategies to improve clinical effectiveness and implementation of anti-SARS-CoV-2 neutralizing antibodies.

The five monoclonal antibody therapies that received EUA by the FDA and/or EMA include two antibody monotherapies and three combination therapies (Table 1). The two monotherapies are developed by GlaxoSmithKline (GSK) and Vir Biotechnology [sotrovimab (S309)] and by Celltrion Healthcare [regdanvimab (CT-P59)]. The combination therapies are developed by Eli Lilly Company [bamlanivimab (LY-COV555) with etesevimab (LY-COV016)], by Regeneron Pharmaceuticals [REGEN-COV2, casirivimab (REGN-10933) with imdevimab (REGN-10987)] and by Astrazeneca [Evusheld, cilgavimab (AZD1061) and tixagevimab (AZD8895)]. Figure 1 shows the binding of these anti-SARS-CoV-2 monoclonal antibody therapies against the RBD of the S protein. In placebo-controlled trials, these antibody therapies reduced the risk of hospitalization and/or death, induced a more rapid decline in viral load, and shortened the time to symptom resolution (44–47). However, there are two major challenges that impact the widespread use of antibody therapies and that are the subjects of this review (Figure 2). First, novel variants of SARS-CoV-2 dominate the ongoing pandemic. The WHO defines new lineages of SARS-CoV-2 as variants of concern (VOCs) in case of higher transmissibility, increased virulence or changes in clinical presentation and/or decreased effectiveness of public health and social measures or available diagnostics, vaccines and therapeutics (48). Following this definition, the Alpha (Pango nomenclature B.1.1.7), Beta (B.1.351), Gamma (P1, B.1.1.28), Delta (B.1.617.2) and Omicron (B.1.1.529) variants were defined as VOCs. As expected, the introduction of VOCs led to an increase in the frequency of re-infection and vaccination breakthrough infections (49, 50). This may be explained by the reduced serum binding and neutralization titers of post-vaccination and convalescent sera against het VOCs compared to the ancestral strain (51, 52). Given this viral escape from naturally and vaccination induced antibodies, the question is to what extent the effectivity of monoclonal antibodies is affected by viral escape. Therefore, this review discusses to what extent these SARS-CoV-2 variants influence the efficacy of the clinically-approved monoclonal antibodies and provide alternative strategies to broaden and enhance antibody activity. Secondly, the main route of antibody administration is intravenously, which limits its usage to a hospital setting. Therefore, strategies which may lead to broader clinical applicability of antibodies against SARS-CoV-2 are also discussed.

**Table 1:** Impact of variants of concern (VOCs) on clinically approved anti-SARS-CoV-2 antibody neutralization titers.

**Anti-SARS-CoV-2 neutralizing antibodies**	**EUA** **as of 01-2022**		**Ancestral strain neutralization**	**Fold change of neutralization titers median (range)**						**References**
	**FDA**	**EMA**	**IC_**50**_ ng/mL**	**Alpha** **B.1.1.7**	**Beta** **B.1.351**	**Gamma** **P1**	**Delta** **B.1.617.2**	**Omicron** **B.1.1.529**		
								**Ancestral**	**Delta**	
**Bamlanivimab** (Ly-CoV555)	No	No	5.0 ng/mL	−1.25 (−1.7 to 2)	>1,000	>1,000	>1,000	>1,000	>1,000	(20–34)
**Etesevimab** (Ly-Cov016, CB6)	No	No	46 ng/mL	13.7 (−1.4 to 54)	>1,000	>1,000	1 (−2 to 1)	>1,000	>1,000	(22, 24, 25, 27–32, 34)
**Bamlanivimab** + **etesevimab**	Yes	WD	7.9 ng/mL	1.3	>1,000	NA	NA	>1,000	>1,000	(25, 27, 30–32)
**Casivirimab** (REGN-10933)	NA	NA	3.2 ng/mL	1,1 (−2.5 to 1.6)	64 (7 to >1,000)	217 (11 to 749)	1 (−1.7 to 19)	630 to >1,000	>1,000	(20–32, 34–37)
**Imdevimab** (REGN-10987)	NA	NA	5.6 ng/mL	−0.3 (−2.1 to 1)	1 (−3 to 9)	−1.7 (−4.3 to 4)	1 (1 to >1,000)	>1,000	>1,000	(20–32, 34–37)
**Ronapreve** (REGN-10933+REGN-10987)	Yes	Yes	1.6 ng/mL	1.6 (1 to 6.3)	1.0 (−1.3 to 20)	1.7 to 6.3	2.5 to 12	501 to >1,000	>1,000 (750 to >1,000)	(20, 21, 24–27, 30–32, 35–38)
**Sotrovimab** (S309)	Yes	Yes	90 ng/mL	1.4 (1.3 to 3.1)	−1.5 (−1.6 to −1.2)	−3.1 (−8.7 to 1.1)	1.3 to 3.5	3.1 (2.9 to 5.0)	3	(20, 21, 24, 25, 29–31, 34, 39)
**Regdanivimab** (CT-P59)	No	Yes	3.5 ng/mL	−5	4	138	183	>1,000	>1,000	(30, 31, 40, 41)
**Tixagevimab** (AZD8895)	NA	NA	4.0 ng/mL	1.5 (−3 to 2)	6.3 (3.5 to 14)	12 (1 to 12)	−2 to 2	>1,000	>1,000	(24, 25, 30, 31, 34, 39, 42)
**Cilgavimab** (AZD1061)	NA	NA	8.1 ng/mL	−2.8 (−3 to −2)	−1.3 (−3 to 1.5)	−2.8 (−4 to 1)	1.5 to 2	324 to >1,000	58	(24, 25, 30, 31, 34, 39, 42)
**Evusheld** (AZD7442) (AZD8895+AZD10661)	Yes	Yes	3.9 ng/mL	1 (1 to 1)	3.8 (3.8 to 10)	2.0	−1.3	110	198	(25, 30, 31, 39, 42)

*The neutralization titers are expressed as the antibody concentration at which the infectivity is inhibited by 50% (IC_50_). The impact is expressed as the fold-change in neutralization titers against the VOCs compared to the ancestral strain (Wuhan-Hu-1 or D614G) neutralization. For Omicron, this fold-change is calculated against the ancestral and the Delta variant. A negative (–) fold-change neutralization titer indicates an improvement of neutralization potency. NA, not available. WD, withdrawn from rolling review*.

### Monoclonal Antibodies Neutralize the Variants of Concern Less Potent

We systematically searched in PubMed for available peer-reviewed studies describing the ability of therapeutic monoclonal antibodies to neutralize VOCs. These titers were compared to the neutralization titers against the Wuhan-Hu-1 or D614G variant that caused the initial epidemic. Both strains are referred to as ancestral strain in the text hereafter. The search was last performed on January 14 2022 and is added to the (Supplementary Material 1) together with a flow-chart listing the number of studies (Supplementary Figure 1).

The Alpha variant was the first VOC to emerge at the end of 2020. This VOC is characterized by a higher transmissibility compared to the ancestral strain and contains one mutation in the RBD at residue N501 (N501Y) (48). This mutation does not lead to a substantial reduction in neutralization titers of the studied monoclonal antibodies (Table 1, Figure 1B) (20–26, 35, 36, 39, 40, 53).

The Beta and Gamma variants do cause a substantial reduction in neutralization titers of several of the clinically approved monoclonal SARS-CoV-2 antibody therapies (20–22, 24–27, 35–37, 39, 40, 42). This reduced neutralization potency of bamlanivimab, etesevimab and casivirimab against the Beta and Gamma variant are predominantly caused by two mutations in the RBM (25, 35, 36, 54–59). These mutations are located at residue E484 (E484K, present in both Beta and Gamma variant) and K417 (K417T present in Beta variant, and K417N present in Gamma variant) (Figure 1B) (48). Accordingly, the combination of bamlanivimab and etesevimab in the Eli-Lilly cocktail is not effective against the Beta and Gamma variants (20, 21, 25). In addition, multiple case-reports reported within host-viral evolution that led to viral escape mutations (E484K, E484Q and Q493R) in immunocompromised COVID-19 patients that were treated with bamlanivimab monotherapy (60–62). These data forced the EMA and FDA to revoke bamlanivimab as a monotherapy (63) and the FDA further limited the usage of the Eli Lilly cocktail for countries where the combined frequency of the Beta and Gamma variants is <5% of circulating stains (64). In contrast, in the Ronapreve cocktail, imdevimab averts the reduced neutralization potency of casivirimab resulting in comparable neutralization titers compared to imdevimab monotherapy (21, 25–27, 35, 36). This illustrates the benefit of using a combination of antibodies compared to a monotherapy, especially when two antibodies are able to bind simultaneously to the RBD but target different epitopes within the RBD (65). However, in case of a combining antibodies that have partially overlapping binding epitopes, mutations in these epitopes that are shared do not necessarily impact the antibody binding to the same extent (42). This is the result of the unique binding property of an antibody, making different epitopes crucial for its attachment. This makes it important to consider the contribution of these shared epitopes to the binding of both antibodies to minimize impact of a single mutation on the efficacy of the combination therapy.

The Beta and Gamma variant did cause a substantial amount of SARS-CoV-2 infections, but never became the dominant variant worldwide. The Delta variant became the most frequently detected strain since the first isolation in late 2020. Originally, the Delta variant did not harbor either the E484K or K417N/T mutation but contained other mutations that impacted antibody recognition (T478K and L452R) and transmissibility (P681R) (Figure 1B) (66). Bamlanivimab lost all neutralization potency against this variant (22, 23, 28–30). However, as etesevimab retains its potency, it is expected that the cocktail remains as effective against the Delta variant as etesevimab monotherapy (28, 29, 66).

Only a few months after the identification of the Delta variant, the Omicron variant emerged in late November 2021 and rapidly became the dominant strain globally. This variant harbors an unusual high number of mutations in the spike protein of which 15 are located in the RBD (Figure 1B) (48). These mutations are located in residues that are known to impact antibody recognition (e.g. K417N, E484A, and T478K) and increase transmissibility (e.g., H69/V70, N501Y and P681H). Almost all previously described clinically approved monoclonal antibodies were substantially impaired to neutralize this VOC (Table 1). Only sotrovimab and Evusheld remain to have some neutralization activity against the Omicron variant. This is especially the case for sotrovimab with only a median 3-fold reduction in neutralization titers (24, 30–32). The binding site of sotrovimab lays outside the RBM, which likely explains the preserved binding properties of this antibody. There are, however, two mutations present in the Omicron variant that fall within the binding epitope of sotrovimab (N440K and G339D), but their contribution to the loss of neutralization remains not fully understood (32).

Taken together, the emerging VOCs heavily influences the neutralization potency of the currently clinical approved monoclonal antibodies. Especially the Omicron variant is resistant against the majority of these antibody therapies. This calls for strategies to broaden the antibody activity. Encouraged by the retained neutralization of sotrovimab against all VOCs, we first discuss other antibody epitopes on the S protein besides the RBD.

### Targeting the NTD and S2-Subunit of the SARS-CoV-2 S Protein

The RBD is the part of the SARS-CoV-2 S protein that is most prone for mutations, as changes in this region could lead to an increased affinity to the ACE-2 receptor and/or escape from pre-existing immunity. In Influenza and HIV-1, regions on the viruses that are less prone to mutate are extensively explored to come closer to a universal therapy (67–69). Compared to the RBD, these more conserved regions of the S protein include the NTD and S2-subunit.

The S2-subunit is the most conserved part of SARS-CoV-2 S protein, a characteristic shared amongst other members of the *Sarbecovirus* family. The sequence identity of the S proteins of SARS-CoV-2 and SARS-CoV is approximately 75%, but increases to 90% for the S2-subunit (70). This sequence identity is further illustrated by the observation that the S2-subunit is the main target for serum IgG cross-reactivity against other members of the *Sarbecovirus* family following SARS-CoV-2 infection and vaccination (71). The S2-subunit contains two heptad motifs and the fusion peptide (10, 11). These domains promote cell membrane fusion by enabling the switch from a pre- to postfusion state after S1/S2-subunit dissociation upon ACE-2 receptor binding (11, 72). Although most S2-targeting antibodies are non-neutralizing, a few neutralizing antibodies are identified to date (73–76). These neutralizing antibodies are thought to inhibit viral infection by preventing the conformation change of the S protein either by locking the S protein in an intermediate conformation, by preventing S1 and S2-subunit cleavage, by targeting the fusion peptide or by stabilizing its pre-fusion conformation (73). One example of an S2-targeting neutralizing antibody is CV3-25, which neutralizes the ancestral strain with a 50% inhibitory concentration (IC_50_) of 120–340 ng/ml (73, 77). This is approximately 100-fold less potent compared to best-in-class RBD-targeting antibodies, and up to 3-fold less potent as sotrovimab to neutralize the ancestral strain (Table 1). A lower neutralization potency makes an antibody less suitable for clinical usage due to the higher amount of protein needed for the same protective or therapeutic effect. On the other hand, this antibody does not only remain fully potent against the Alpha, Beta, Gamma and Delta variant of SARS-CoV-2, it also cross-neutralizes the SARS-CoV-1 virus (73, 77). This illustrates that broad antibody activity often comes at the cost of neutralizing potency.

Another domain of the S protein that has gained less interest is the N-terminal domain (NTD). The NTD is part of the S1-subunit and is highly shielded by *N*-linked glycans. This glycan shield covers potential neutralizing antibody targets and thereby leads to a limited immunogenicity. Although the exact function of the NTD remains unknown, it has been shown that NTD is involved in the viral entry by binding co-receptors on the human target cell, such as the L-SIGN/DC-SIGN receptors. These receptors are pattern recognition receptors that belong to the C-type lectin family (78). NTD-binding antibodies may contribute to the immunity against SARS-CoV-2 infection by either disrupting this interaction with co-receptors, or by preventing the ACE-2 and RBM interaction by sterical hindrance and/or by executing Fc effector functions (79–83). Because their mechanism of action differs from most RBD-targeting antibodies, they may be a relevant contribution to a cocktail with RBD-targeting antibodies to broaden its activity. The NTD is divided in six antigenic sites, but the majority of neutralizing antibodies target the so called NTD-supersite (73, 79, 80, 82, 83). One of the most potent neutralizing NTD-targeting antibodies, S2X333, neutralizes the SARS-CoV-2 pseudoviruses with an IC_50_ of 2 ng/ml (79). This neutralization capacity is comparable to the neutralization capacity of the most potent clinically approved monoclonal antibodies therapies (Table 1). In addition, S2X333 effectively prevented SARS-CoV-2 infection in a Syrian hamster model. The NTD domain is less conserved compared to the S2-subunit, but fewer mutations between SARS-CoV-2 variants are identified in this region compared to the RBD. However, all VOCs emerged to date harbor mutations in the NTD and especially in the NTD-supersite (22, 79, 84). This increases the likelihood that the neutralizing potency of most NTD-targeting antibodies will be negatively affected by the VOCs, although this remains to be studied for S2X333 (31).

### Targeting Multiple Sites of Vulnerability on the S Protein Simultaneously

The neutralization potency of the monoclonal antibody combination therapy Ronapreve (REGN-COV) and Evusheld (AZD7442) are not substantially influenced against any VOCs, except the Omicron variant (Table 1). The retained neutralizing potency shows the benefit of targeting multiple sites on the S protein simultenously. Following this principle, a valuable alternative for antibody cocktails may include bi-specific of multi-valent antibodies.

A classical Y-shaped IgG molecule consists of two identical heavy and light chains and therefore contains two identical antigen binding sites (Figure 2). A bi-specific antibody consists of two, artifically linked, different antigen binding sites into one molecule and can thereby bind two different epitopes at the same time (85). Different antigen binding sites on the same molecule increases the likelyhood of bridging two epitopes on the same antigen and thereby increasing avidity. This results in an additive or synergistic effect which cannot be achived by simply combining the two parental IgG monoclonal antibodies (85). From a developmental and logistic point of view, another advantage of bi-specific antibodies is their cost-effective production, because only one molecule needs to be tested for safety and efficacy in (pre-)clinical trials (86). Based on the position of the antigen binding sites related to the fragment crystallizable (Fc) domain, the “tail” of an IgG antibody, the family of IgG-like bi-specifics can be divided into asymmetric or symmetric constructs (Figure 2). In an asymetric construct, the heavy and light chains of the two parental antibodies are heterologous matched, which results in two different variable regions on both arms of the IgG-like antibody. The proces of heterologous chain matching results in a high rate of incorrectly matched side products. This chain mismatching is therefore one of the major challenges to overcome in developing bi-specific IgG like antibodies. To promote heterologous chain matching, numerous strategies are under development (85). In a symmetric format, the variable regions are coupled by a linker to the same arm of thet IgG or to the Fc domain, resulting in four variable regions per antibody (Figure 2) (85). Besides the difference in amount of variable regions, another difference between the asymmetric and symmetric constructs is the distance between the variable regions. In an asymmetric construct, this distance is fixed as it depends on the hinge length of the antibody. In constrast, the distance between the variable regions of a symmetric conctruct is depending on the length of the linker that connects the variable region to the antibody. The binding to an antigen can therefore be optimized by changing the length of the linker.

It was in 2014 that the FDA approved for the first time a bi-specific antibody for clinical usage in humans (87). This approval of blinatumomab for the treatment of B-cell acute lymphoblastic leukemia inspired researchers to explore the possibilities for using bi-specific antibodies in infectious diseases (88). In the field of HIV-1 and influenza, various bi-specific antibodies showed a reduction of viral load and protection against disease in pre-clinical studies (86, 89), driving these constructs into phase-I human clinical trials (90). Bi-specific antibodies have also been developed against SARS-CoV-2. The majority of these bi-specific antibodies target two different epitopes within the RBD (91–95). One example of an assymetric RBD-targeting bi-specific antibody is CoV-X2, which neutralizes the ancestral strain with a comparable potency of imdevimab or Evusheld (IC_50_ of 5.8 ng/ml) (**Tabel 1**) (91). This bi-specific antibody remained effective against pseudoviruses that contain escape mutations that are induced by the parental monoclonal antibodies. This illustrates the complementary functionality of this bi-specific antibody. However, although very potent in neutralizing the Alpha variant, CoV-X2 was less effective in neutralizing the Beta variant (IC_50_ of 191 ng/ml) (91). In addition to this RBD-targeting bi-specific antibody, Cho at al. developed a symmetric bi-specific antibody, CV503_664_GS, that targeted both the NTD and RBD domain. A linker of 15 amino acids is used to couple the variable regions to the antibody and enabled bridging of relatively distinct epitopes (NTD and RBD). This resulted in a 100-fold increase in neutralizing potency against the ancestral strain (IC_50_ of 4.1 ng/ml) compared to the combination regimen of the parental monoclonal antibodies (96). This bi-specific antibody retained its high neutralizing potency against the Alpha, Beta, Gamma and Delta variant. To the best of our knowledge, no data is yet published on the neutralizing potency of CV503_664_GS against the Omicron variant.

In summary, the current SARS-CoV-2 bi-specific antibodies are at least as potent as the clinical approved monoclonal antibody therapies in neutralizing the ancestral SARS-CoV-2 strain, and may therefore render an interesting candidate for future antibody therapies to combat VOCs (91, 95, 96). However, as no bi-specific antibodies against SARS-CoV-2 are yet in human clinical trial, it is unlikely that bi-specific antibodies will soon become a treatment option against COVID-19.

### The Role of the Fc-Domain Engineering on the Effectiveness of Antibodies

In addition to the two IgG-like bi-specific SARS-CoV-2 antibodies that we described in the previous paragraph, there are also bi-specific and multivalent antibodies developed against SARS-CoV-2 which lack the Fc domain (94, 95, 97). The Fc domain of an antibody is important for its solubility, stability and increases the antibody half-life. Besides these qualities, the Fc domain is also involved in the recruitment and activation of innate immune cells via binding to Fc gamma receptors (FcγRs). These Fc mediated effector functions include the induction of antibody dependent cellular cytotoxicity (ADCC), cellular phagocytosis (ADCP), cellular trogocytosis (ADCT) and complement deposition (ADCD) (98, 99). Mutations that lead into an enhanced, a reduced or even a loss-of-function (e.g., Fc-dead) of the Fc domain are often used to quantify the contribution of Fc effector functions on the effectiveness antibodies (99). In case Fc effector functions are desired, monoclonal antibodies are usually produced with an IgG1 Fc domain. The impact of the Fc domain on the effectiveness of SARS-CoV-2 antibodies is still under debate and seems to depend on many factors.

Most studies agree that anti-SARS-CoV-2 antibodies with intact Fc effector functions are beneficial over Fc-dead antibodies and contribute to the prevention of weight loss, reduction of viral load and prevent death in small animal models, especially when used a treatment (98, 100–102). For example, Yamin et al. studied the influence of Fc domain engineering on the effectiveness of the Ronapreve cocktail (casirivimab with imdevimab) to prevent weight loss and death in mice (102). The cocktail was not effective as treatment when harboring loss-of-function mutations in its Fc domain. In addition, the insertion of mutations that enhanced specific Fc effector functions resulted in a better protection against weight loss in this mouse model compared to the original IgG1 antibody. This improved effectiveness due to Fc engineering is also demonstrated for the previously mentioned S2-targeting antibody CV3-25 (100, 103). An increased effectiveness substantially reduces the therapeutic and prophylactic dose required for the same protection, which is very relevant in times of scarcity (102).

However, caution is needed when it comes to enhancing the Fc effector function of an antibody selected for (pre-)clinical usage. There exists a thin line between the desired pro-inflammatory activation of the immune system by the Fc domain, and the threshold for over-activation that may result in a cytokine storm (104). This delicate balance is illustrated by the recent finding that higher levels of IgG against the S protein with enhanced Fc effector functions are detected in sera of hospitalized COVID-19 patients compared to asymptomatic and non-hospitalized patients (103–106). These pro-inflammatory antibodies lack the core fucose residue in the Fc domain which enhances the affinity to the FcyRIIIa and promotes activation of myeloid cells. It is thought that these antibodies contribute to the hyperinflammatory syndrome observed in these patients (104, 105). This hyperinflammatory syndrome is reflected in the ARDS that occurs in severe COVID-19 and that is caused by pulmonary edema due to inflammation induced endothelial barrier disruption and thrombosis (105, 107).

So, in small animal models these pro-inflammatory antibodies seem to benefit the protection against disease, while they are also associated with severe COVID-19 in humans. A possible explanation for this discrepancy may be the timing of studying the effect of antibody Fc effector functions on disease progression. The innate immune system has a prominent role at the start of an infection as it acts as one of the first defense mechanisms against pathogens (108). Keeping this in mind, antibodies with an optimized Fc effector function could be beneficial when used as prophylactic or as treatment shortly after onset of symptoms. On the other hand, the innate immune system should be in balance with the adaptive immune system around the time of seroconversion. Administering antibodies with an enhanced Fc effector function in this stage of COVID-19 may therefore lead to over-activation of the innate immune system. In addition, the role of antibodies, regardless of Fc engineering, in the treatment against COVID-19 after seroconversion is also under debate (109).

The added value of Fc engineering may also depend on the neutralization potency of the antibody, as no influence of Fc engineering is observed when the parental antibody has an extremely high neutralization potency (110). This may also explain the need for maintained Fc effector functions of some less potent NTD-targeting antibodies (IC_50_ 501 and 119 ng/ml) in order to prevent disease (83), while this is not the case for more potent neutralizing NTD-targeting antibodies (111). In addition, the influence of Fc engineering may also depend on the given dose, as was shown for the Ronapreve cocktail where the impact of Fc engineering became negligible when the cocktail was administered in higher dose (100).

Altogether, Fc domain engineering may improve the effectiveness of SARS-CoV-2 monoclonal antibodies, which should encourage further research. However, the added value of Fc engineering seems to depend on many factors, including the timing of administration since symptom onset, the usage of antibody as prophylaxis or treatment and the neutralization potency of the parental antibody.

### Broadening Clinical Usability by Half-Life Extension and Non-intravenous Administration of Antibodies

Additionally, there are two pharmacokinetic challenges that impact the broad roll-out of monoclonal antibody therapies to prevent or treat COVID-19. The first challenge includes the relatively short half-life (21 days) of human antibodies, which currently dictates a monthly injection when used as prophylactic. The Fc domain of an antibody determines the half-life of the antibody as the large size prevents rapid renal clearance (112). The elimination of antibodies predominantly occurs throughout the human body by intracellular catabolism by lysosomal degradation. The clearance of antibodies by immune cells after Fc domain binding to FcγRs plays only a minor role (112). However, antibodies have a long half-life compared to other proteins with similar molecular weight, as IgG antibodies are rescued from lysosomal degradation by Ph-dependent binding of the Fc domain to the neonatal Fc receptor (FcRn) and recycled back to the cellular membrane. The clinically approved monoclonal antibody cocktail Evusheld (AZD7442) contains two long-acting antibodies (tixagevimab with cilgavimab) which both contain Fc domain mutations that lead to an increased affinity to the FcRn receptor (113). This YTE half-life extension technology includes M252Y/S254T/T256E (YTE) mutations in the Fc domain, resulting in a pH-dependent higher affinity to the FcRn and reduced ADCC effector function of the Fc domain (114). Due to this half-life extension, their effectiveness as a pre-exposure prophylaxis to prevent symptomatic COVID-19 is expected to last up to 12 months after administration (115).

The second challenge that hinder widespread utilization of monoclonal antibodies includes the route of antibody administration. In general, monoclonal antibodies against COVID-19 are administered intravenously, which limits their usage to a hospital setting. However, Evusheld is also the first monoclonal antibody regime approved for intramuscular injection. In addition, preliminary results reported by GSK show that the safety and effectiveness of sotrovimab is similar when administered intramuscularly or intravenously (116). The possibility to administer antibodies intramuscularly is an important step toward the possibility of widespread and early treatment of COVID-19.

Administering antibodies via inhalation may be an interesting alternative for systemic application. As SARS-CoV-2 enters the human body via the respiratory tract, direct administration of antibodies via inhalation or a nasal spray may tackle the virus even before it enters the human body. Especially when realizing that the antibody titers in the respiratory tract are approximately 15% of the plasma concentration, direct administration via inhalation seems highly efficient over systemic administration (117). The respiratory administration of antibodies have been shown to be safe, effective and feasible against other respiratory diseases (118). Also for SARS-CoV-2, there are studies showing that inhaled antibodies are safe and effective in small animal models to prevent and treat COVID-19 (119). One of the major challenge is, however, the instability of antibodies due to nebulization (120). Nanobodies (Nb) can be a useful alternative for human monoclonal antibodies due to their high thermal stability and solubility (121). Nanobodies are variable heavy chain domains (VHHs) derived from heavy chain only immunoglobulins produced by Camelids. Compared to an IgG-like antibody, nanobodies are much smaller (15kDa instead of 150kDa) as they lack an Fc domain. Their small size enables improved tissue penetration and the recognition of epitopes that are largely inaccessible for human antibodies, which makes them also an interesting additive to the arsenal of human SARS-CoV-2 S protein targeting IgG antibodies (122). Most VHHs against SARS-CoV-2 are isolated from SARS-CoV-2 S protein immunized Camelids (95, 122). One of the most potent SARS-CoV-2 targeting nanobodies discovered today is a tri-specific nanobody that targets twice the same RBD epitope (Nb_15_) and also binds to the human serum albumin (Nb–_h_) (95). The latter helps to overcome the short half-life of nanobodies due to the absence of an Fc domain. This Nb_15_-Nb_H_-Nb_15_ nanobody has a neutralizing potency that exceeds the potency of any current clinically approved monoclonal antibody therapy (IC_50_ of 0.4ng/ml) and is effective for both prophylactic and therapeutic purposes against SARS-CoV-2 infection in mice when administered intranasally. It neutralizes the Alpha and Delta variant with comparable neutralizing potency (IC_50_ of 0.26 and 5.2 ng/ml, respectively) but fails to neutralize the Beta and Gamma variant. In summary, inhaled antibodies may be a valuable alternative for systemic antibody administration, although its safety and efficacy against COVID-19 should first be tested in non-human primates before continuing with phase-I clinical trials.

## Conclusion

In the beginning of the COVID-19 pandemic, research efforts were mostly focused on the isolation of highly potent neutralizing antibodies against SARS-CoV-2. However, with the substantial reduced neutralization of antibodies against the emergence of VOCs to date, there is an urgent need for a renewed balance between neutralizing antibody potency and breadth. This review discussed multiple promising strategies to broaden antibody activity against SARS-CoV-2. Seeing the ongoing COVID-19 pandemic against the light of the described promising avenues to improve antibody therapies, underpins the urgency to further develop these compounds and start clinical studies soon.

## Author Contributions

KS conceptualized, wrote, and edited the manuscript. MG and GB conceptualized, contributed writing to, and edited the manuscript. ST conceptualized sections and edited the manuscript. All authors reviewed and approved the final manuscript.

## Conflict of Interest

The authors declare that the research was conducted in the absence of any commercial or financial relationships that could be construed as a potential conflict of interest.

## Publisher's Note

All claims expressed in this article are solely those of the authors and do not necessarily represent those of their affiliated organizations, or those of the publisher, the editors and the reviewers. Any product that may be evaluated in this article, or claim that may be made by its manufacturer, is not guaranteed or endorsed by the publisher.

## Supplementary Material

Figure S1 Flow-chart of PubMed Search.
