# Supplementary figures and images for: Optimization of Anti-SARS-CoV-2 Neutralizing Antibody Therapies: Roadmap to Improve Clinical Effectiveness and Implementation
Source: Front Med Technol. 2022 Mar 28;4:867982. doi: 10.3389/fmedt.2022.867982 (PMC8996231; doi:10.3389/fmedt.2022.867982)

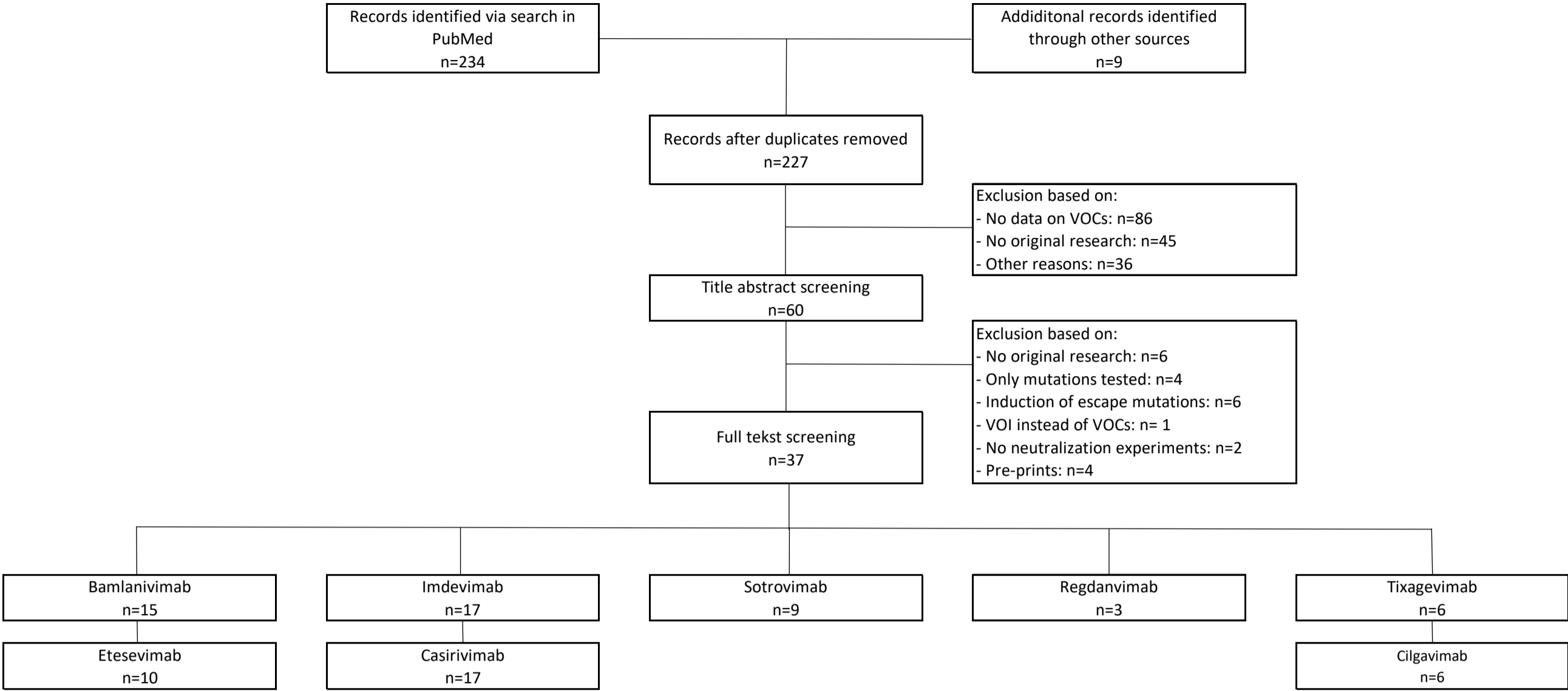

Supplement: Figure S1 — Flow-chart of PubMed Search. [file Data_Sheet_1.PDF]
