# Supplementary material for: Optimization of Anti-SARS-CoV-2 Neutralizing Antibody Therapies: Roadmap to Improve Clinical Effectiveness and Implementation
Source: Front Med Technol. 2022 Mar 28;4:867982. doi: 10.3389/fmedt.2022.867982 (PMC8996231; doi:10.3389/fmedt.2022.867982)
Supplement: Supplementary file 2 [file Data_Sheet_2.PDF]

Supplementary Material 1. PubMed search on the impact of SARS-CoV-2 variants of concern (VOCs) on the clinically approved neutralizing antibody therapies.

((((((((SARS-CoV-2[Title/Abstract]) OR (SARS Coronavirus 2[Title/Abstract])) OR (COVID-19[Title/Abstract])) OR (COVID19[Title/Abstract])) OR (2019-nCoV[Title/Abstract])) OR (2019 Novel Coronavirus Disease[Title/Abstract])) OR (Coronavirus Disease 2019[Title/Abstract])) OR ("covid 19"[MeSH Terms])) OR (SARS-CoV-2[MeSH Terms])

AND

((((((((((((((((((((((((Eli Lilly[Title/Abstract]) OR (Bamlanivimab[Title/Abstract])) OR (LY-CoV555[Title/Abstract])) OR (Etesevimab[Title/Abstract])) OR (LY-CoV016[Title/Abstract])) OR (JS016[Title/Abstract])) OR (CB6[Title/Abstract])) OR (REGN-COV2[Title/Abstract])) OR (Regeneron[Title/Abstract])) OR (Casirivimab[Title/Abstract])) OR (REGN10933[Title/Abstract])) OR (Imdevimab[Title/Abstract])) OR (REGN10987[Title/Abstract])) OR (Sotrovimab[Title/Abstract])) OR (VIR-7831[Title/Abstract])) OR (GSK4182136[Title/Abstract])) OR (Xevudy[Title/Abstract])) OR (S309[Title/Abstract])) OR (Regdanvimab[Title/Abstract])) OR (CT-P59[Title/Abstract])) OR (Regkirona[Title/Abstract])) OR (AZD7442[Title/Abstract])) OR (AZD8895[Title/Abstract])) OR (tixagevimab[Title/Abstract])) OR (AZD1061[Title/Abstract])) OR (cilgavimab[Title/Abstract])

AND

(((((((Neutrali*[Title/Abstract]) OR (Bind*[Title/Abstract])) OR (Kd[Title/Abstract])) OR (Affinity[Title/Abstract])) OR (Dissociat*[Title/Abstract])) OR (Antibodies, Neutralizing[MeSH Terms])) OR (Antibodies, Blocking[MeSH Terms])) OR (Antibody affinity[MeSH Terms]) OR (((((((((((Disease severity[Title/Abstract]) OR (Surviv*[Title/Abstract])) OR (Hospital*[Title/Abstract])) OR (Admission[Title/Abstract])) OR (Mortal*[Title/Abstract])) OR (Shed*[Title/Abstract])) OR (Load[Title/Abstract])) OR (Titer[Title/Abstract])) OR (Hospitalization[MeSH Terms])) OR (Mortality[MeSH Terms])) OR (Viral load[MeSH Terms])) OR (Virus shedding[MeSH Terms])
